# Phenylphenalenones and Linear Diarylheptanoid Derivatives Are Biosynthesized via Parallel Routes in *Musella lasiocarpa*, the Chinese Dwarf Banana

**DOI:** 10.1021/acs.orglett.4c01750

**Published:** 2024-06-20

**Authors:** Hui Lyu, Lukas Ernst, Yoko Nakamura, Yu Okamura, Tobias G. Köllner, Katrin Luck, Benye Liu, Yu Chen, Ludger Beerhues, Jonathan Gershenzon, Christian Paetz

**Affiliations:** † NMR/Biosynthesis Group, Max Planck Institute for Chemical Ecology, Jena 07745, Germany; ‡ Technische Universität Braunschweig, Institute of Pharmaceutical Biology, Braunschweig 38106, Germany; § Department of Biochemistry, Max Planck Institute for Chemical Ecology, Jena 07745, Germany; ∥ Department of Natural Product Biosynthesis, Max Planck Institute for Chemical Ecology, Jena 07745, Germany; ⊥ Jiangsu Key Laboratory for the Research and Utilization of Plant Resources, Institute of Botany, Jiangsu Province and Chinese Academy of Sciences (Nanjing Botanical Garden Mem. Sun Yat-Sen), Nanjing 210014, China; # Department of Insect Symbiosis, Max Planck Institute for Chemical Ecology, Jena 07745, Germany

## Abstract

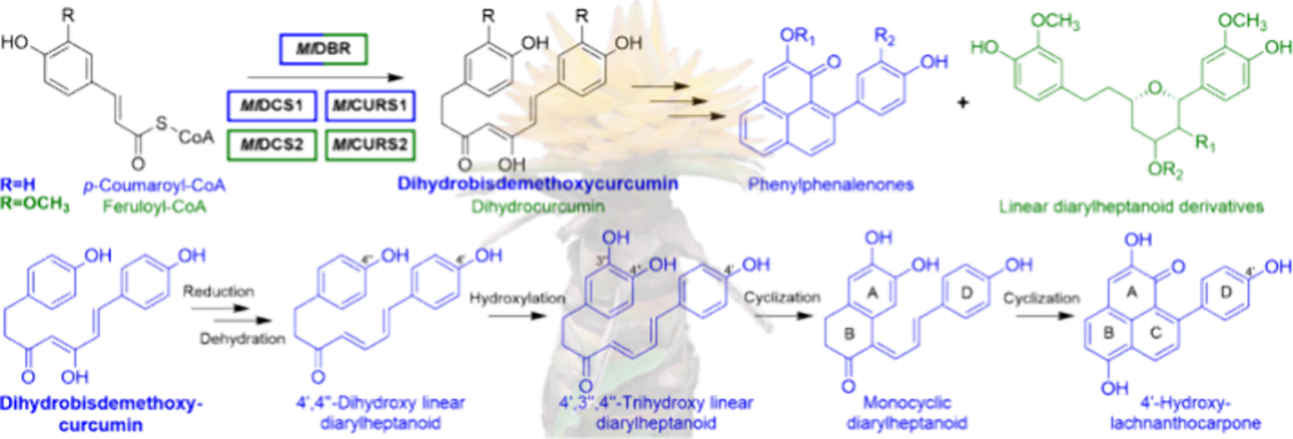

Here, we use transcriptomic data from seeds of *Musella lasiocarpa* to identify five enzymes involved in the formation of dihydrocurcuminoids. Characterization of the substrate specificities of the enzymes reveals two distinct dihydrocurcuminoid pathways leading to phenylphenalenones and linear diarylheptanoid derivatives, the major seed metabolites. Furthermore, we demonstrate the stepwise conversion of dihydrobisdemethoxycurcumin to the phenylphenalenone 4′-hydroxylachnanthocarpone by feeding intermediates to *M. lasiocarpa* root protein extract.

Phenylphenalenones (PPs) are complex polycyclic natural products that play an important role in the chemical defense system of banana and plantain (Musaceae).^1^ Previous studies demonstrated the antipathogen activities of PPs^2−11^ and linked the increased accumulation of PPs to the increased viability of disease-resistant banana varieties.^8−10^ A partial biosynthetic hypothesis for PPs proposed in 1961^12^ was later validated through isotope labeling experiments, leading to the proposed biosynthetic pathway depicted in Scheme 1.^1^ In the initial stage of this biosynthetic scenario, a linear diarylheptanoid (DH) is formed by the successive condensation of two phenylpropanoids with a malonate unit.^13−18^ After the A-ring hydroxylation of the linear DH intermediate,^19,20^ a hypothetical intramolecular Diels–Alder cyclization step yields the PP scaffold.^21−24^ However, most of the intermediates of PP biosynthesis are still unknown and none of the genes involved in the pathway have been identified.

**Scheme 1:**
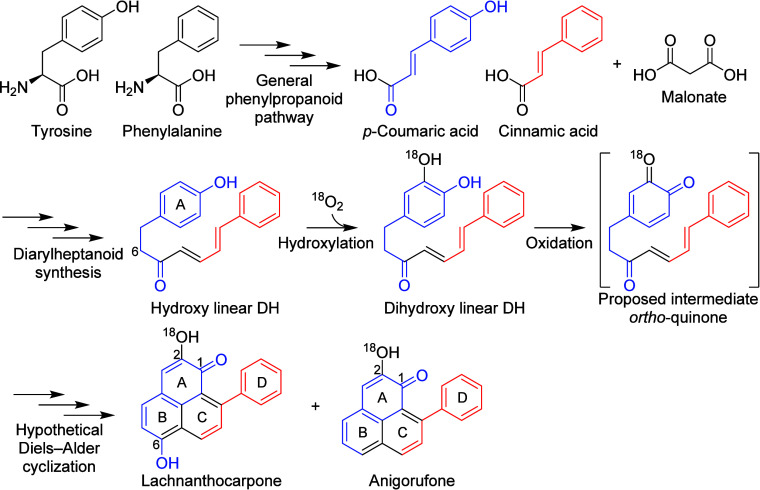
Proposed Biosynthetic Pathway of Phenylphenalenones

In a recent study, we elucidated the structures of various PPs and linear DH derivatives found in the seeds of the Chinese dwarf banana (*Musella lasiocarpa*, Musaceae), which were present at later stages (brown and black seeds) but absent at early stages of development (yellow seeds) (Figure 1A).^25^ Based on their structural features, we hypothesized that the assembly of both compound classes likely shares the initial steps of dihydrocurcuminoid biosynthesis and involves two different starter substrates (*p*-coumaroyl-CoA **1** and feruloyl-CoA **2**; Figure S1). The biosynthesis of curcuminoids has been well studied in *Curcuma longa* and involves the sequential action of the type III polyketide synthases diketide-CoA synthase (DCS) and curcumin synthase (CURS) (Figure S3).^26^

**Figure 1:**
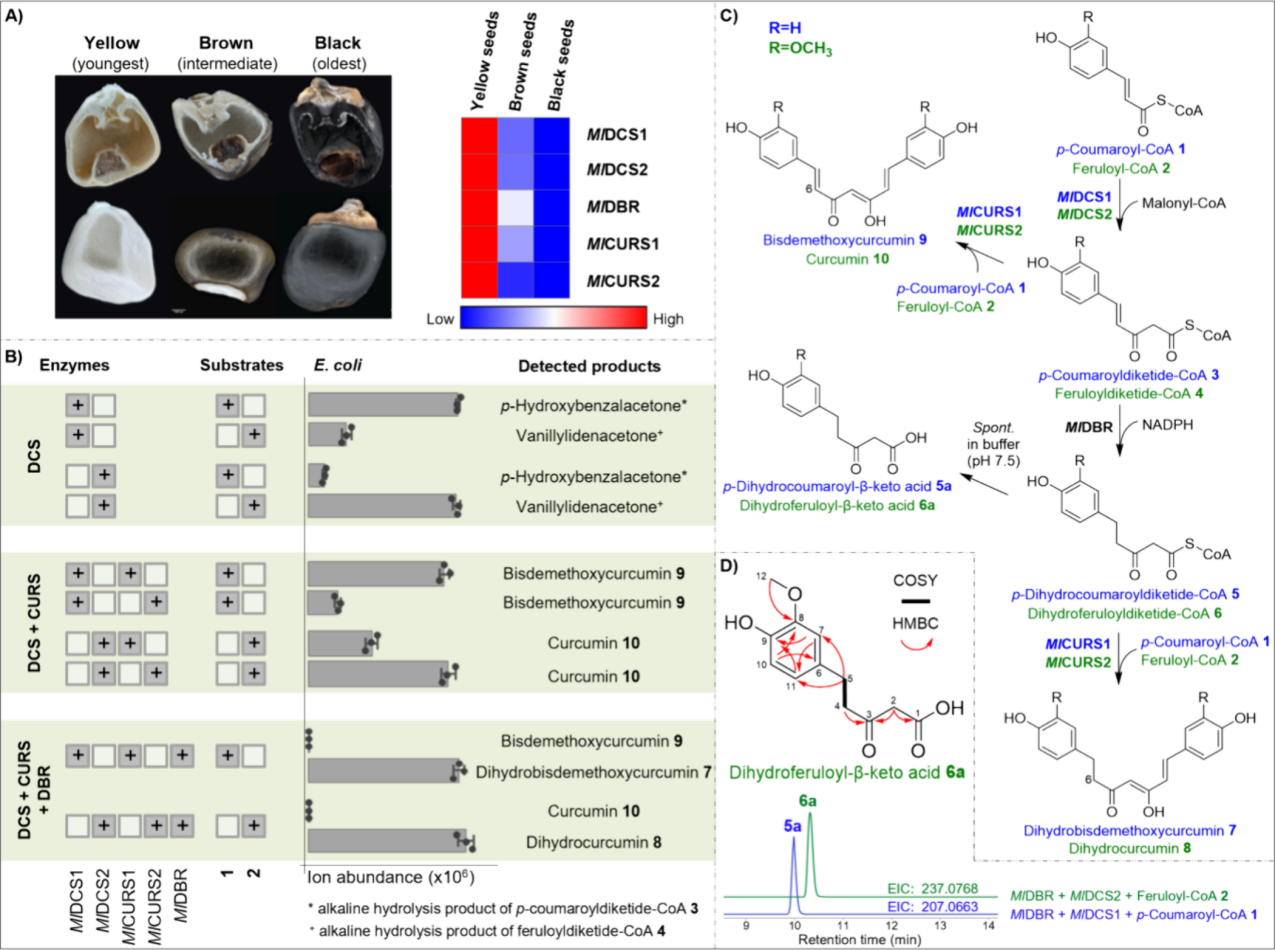
Biosynthesis of dihydrocurcuminoids in *M. lasiocarpa* seeds. (A) Expression profiles of the identified genes representing the FPKM values in the three developmental seed stages. (B) The HRESIMS peak area of products detected in assays using the indicated combinations of purified recombinant enzymes and substrates. Diketide-CoA esters were detected after alkaline hydrolysis. Data are means ± s.e.m. (*n* = 3). (C) The biosynthetic pathway for the formation of dihydrocurcuminoids (**7** and **8**) and curcuminoids (**9** and **10**). (D) Extracted ion chromatograms (EIC) of the spontaneously hydrolyzed dihydrodiketide-CoA products, **5a** and **6a**. Key COSY and HMBC correlations of **6a** are shown above. Compound names in blue, R = H; compound names in green, R = OCH_3_.

Here, we perform a *de novo* transcriptome assembly and compare gene expression across three seed stages. A translated nucleotide search (tBLASTn) using DCS and CURS as query sequences led to the identification of two DCS homologues (*Ml*DCS1 and *Ml*DCS2) and two CURS homologues (*Ml*CURS1 and *Ml*CURS2), which all were highly expressed in yellow seeds (Figure 1A).

The catalytic activities of *Ml*DCS1 and *Ml*DCS2 were established in assays using purified enzymes overexpressed in *Escherichia coli*. Both enzymes accepted various phenylpropanoid-CoA esters as starter substrates and used one molecule of malonyl-CoA as the extender substrate to catalyze the formation of the corresponding diketide-CoAs (Figure S4). The benzalacetone derivatives were detected after alkaline hydrolysis using high-resolution electrospray ionization mass spectrometry (HRESIMS). Product identities were confirmed by comparing their retention times and MS/MS fragmentation patterns with those of commercial standards. Interestingly, the substrate specificities of the two enzymes differed. While *Ml*DCS1 clearly preferred *p*-coumaroyl-CoA **1** as the starter substrate, *Ml*DCS2 favored feruloyl-CoA **2** (Figure 1B). To test the catalytic function of the enzymes in the plant environment, *Nicotiana benthamiana* leaves were transiently transformed via *Agrobacterium*-mediated infiltration. HRESIMS analysis of alkaline-hydrolyzed leaf extracts revealed that plants expressing *Ml*DCS1 exclusively formed *p*-coumaroyldiketide-CoA **3**, while feruloyldiketide-CoA **4** was the only product formed in plants expressing *Ml*DCS2 (Figure S4).

The enzymatic functions of *Ml*CURS1 and *Ml*CURS2 were assessed in co-incubations with purified *Ml*DCS1 or *Ml*DCS2 in the presence of their corresponding preferred starter substrates, **1** and **2**. The two *Ml*CURSs were able to catalyze the decarboxylative condensation of diketide-CoAs **3** or **4** with another molecule of the substrates **1** or **2**, respectively, to yield bisdemethoxycurcumin **9** and curcumin **10**, respectively (Figure 1C). However, *Ml*CURS1 showed higher activity in combination with *Ml*DCS1 and **1**, while *Ml*CURS2 performed better with *Ml*DCS2 and **2** (Figure 1B). These findings were further substantiated through co-infiltration experiments with different combinations of *Ml*DCS and *Ml*CURS in *N. benthamiana* leaves, in which *Ml*CURS1 and *Ml*CURS2 exhibited the same substrate preferences observed in the *in vitro* assays (Figures S5 and S6).

Given that curcuminoids **9** and **10** possess a Δ^6^ double bond, while the putative PP precursors (Scheme 1) and all isolated linear DH derivatives^25^ are saturated at C-6, reduction of the Δ^6^ double bond may be a shared step in the biosynthesis of both PPs and linear DH derivatives (Figure S1). It could occur at either the phenylpropanoid-CoA, diketide-CoA, or curcuminoid stage (Figure S7A). We searched for double bond reductases (DBRs) that could act on double bonds in conjugation with carbonyl functions in the *de novo* transcriptome assembly of *M. lasiocarpa*. A total of nine homologues were annotated as NADPH-dependent 2-alkenal reductases. However, only one of them, named *Ml*DBR, showed an expression pattern similar to those of *Ml*DCS and *Ml*CURS (Figure 1A). The activity of the recombinant enzyme was initially tested against *p*-coumaroyl-CoA **1**, feruloyl-CoA **2**, bisdemethoxycurcumin **9**, and curcumin **10**. However, no conversion was detected for any of the phenylpropanoid-CoA and curcuminoid substrates.

We next tested diketide-CoAs as potential substrates by incubating *Ml*DBR with *Ml*DCS1 or *Ml*DCS2 in the presence of their respective starter substrates **1** and **2**, malonyl-CoA, and NADPH. A mass peak at *m*/*z* 237.0765 [M – H]^−^ (calcd for C_12_H_13_O_5_, 237.0768) was detected in assays comprising *Ml*DBR, *Ml*DCS2, substrate **2**, and NADPH (Figure S7). The product was isolated from large-scale incubations and its structure was elucidated by NMR spectroscopy (Figures S20–S23 and Table S1), revealing the previously undescribed dihydroferuloyl-β-keto acid **6a** (Figure 1D). The corresponding product, *p*-dihydrocoumaroyl-β-keto acid **5a**, was identified in co-incubations containing *Ml*DBR, *Ml*DCS1, substrate **1**, and NADPH through detection of an ion at *m*/*z* 207.0664 [M – H]^−^ (calcd for C_11_H_11_O_4_, 207.0663; Figure S7). The recorded mass was consistent with the loss of a methoxy group from **6a**, and comparison of MS/MS fragmentation patterns further supported this identification (Figure S7C). Spontaneous hydrolysis of dihydroferuloyldiketide-CoA **6** to **6a** in pH 7.5 buffer was expected and underlined by a linear correlation between the formation of **6a** and the incubation time of the assay (from 1 to 10 h; Figure S7D).

The addition of *Ml*DBR to the previously conducted co-incubation assays of *Ml*DCS1/*Ml*CURS1 and *Ml*DCS2/*Ml*CURS2 led to the formation of the expected dihydrocurcuminoid products dihydrobisdemethoxycurcumin **7** and dihydrocurcumin **8**, respectively (Figure 1B and Figure S5). No traces of curcuminoids **9** and **10** were observed in the assays, indicating the efficiency of this reduction step. These results were consistently replicated through co-infiltration experiments in *N. benthamiana* using the same enzyme combinations (Figures S5 and S6). We therefore characterize *Ml*DBR as an unusual diketide-CoA-accepting member of the zinc-independent medium-chain dehydrogenase/reductase superfamily, containing two conserved NADPH-binding motifs AXXGXXG and GXXS (Figure S8).^27^ To the best of our knowledge, no DBR accepting diketide-CoAs as substrates has been reported so far.

The PP skeleton was previously proposed to be formed from 4′,3″,4″-trihydroxy linear DH **12** via a reactive *ortho*-quinone intermediate that undergoes intramolecular Diels–Alder-like cyclization to yield PPs (Figure S9).^29^ Of the intermediates formed in the above reactions, we considered **7** is a likely precursor of **12** because of its ring substitution pattern (Figure 2A). The conversion of **7** to **12** could proceed via reduction and dehydration to the linear DH **11** with an α, β, γ, δ-unsaturated ketone function, followed by hydroxylation on the A ring at the C-3″ position (Figure 2A). To demonstrate this conversion, we employed protein extracts from *M. lasiocarpa*. Due to the difficulties in preparing cell-free homogenates from the hard seeds, we explored roots as a possible alternative. The outermost root layer proved to be another rich source of aromatic metabolites, including detectable amounts of **7** (Figure S10). Incubation of 2′,3′,5′,6′-deuterium-labeled **7** (*d*_4_-**7**) with NADPH in the crude root protein extracts resulted in the appearance of two [M + H]^+^ ions at *m*/*z* 299.1578 and 315.1520, matching the expected masses of *d*_4_-**11** (calcd for C_19_H_15_D_4_O_3_, 299.1580) and *d*_4_-**12** (calcd for C_19_H_15_D_4_O_4_, 315.1529) (peaks a and b; Figure S11B). No activity was observed in control assays lacking NADPH or using heat-inactivated proteins. To determine the identity of **11** and **12**, these compounds were isolated in greater quantities from root methanolic extracts and structural determination was carried out by NMR (Figures S44–S53). The MS/MS spectra of the isolated compounds matched those obtained from the corresponding products **11** and **12** formed when **7** or *d*_4_-**7** was incubated with root protein extract. An additional peak observed on incubation with **7** and *d*_4_-**7** had the same retention time as **12** but was found to be hydroxylated on the other aromatic ring (**12a**) (Figure S11). Since **12a** did not occur in the methanolic plant extract and could not be further converted into corresponding PPs, we suspect it to be an artifact obtained only by feeding potential intermediates to root extracts.

**Figure 2:**
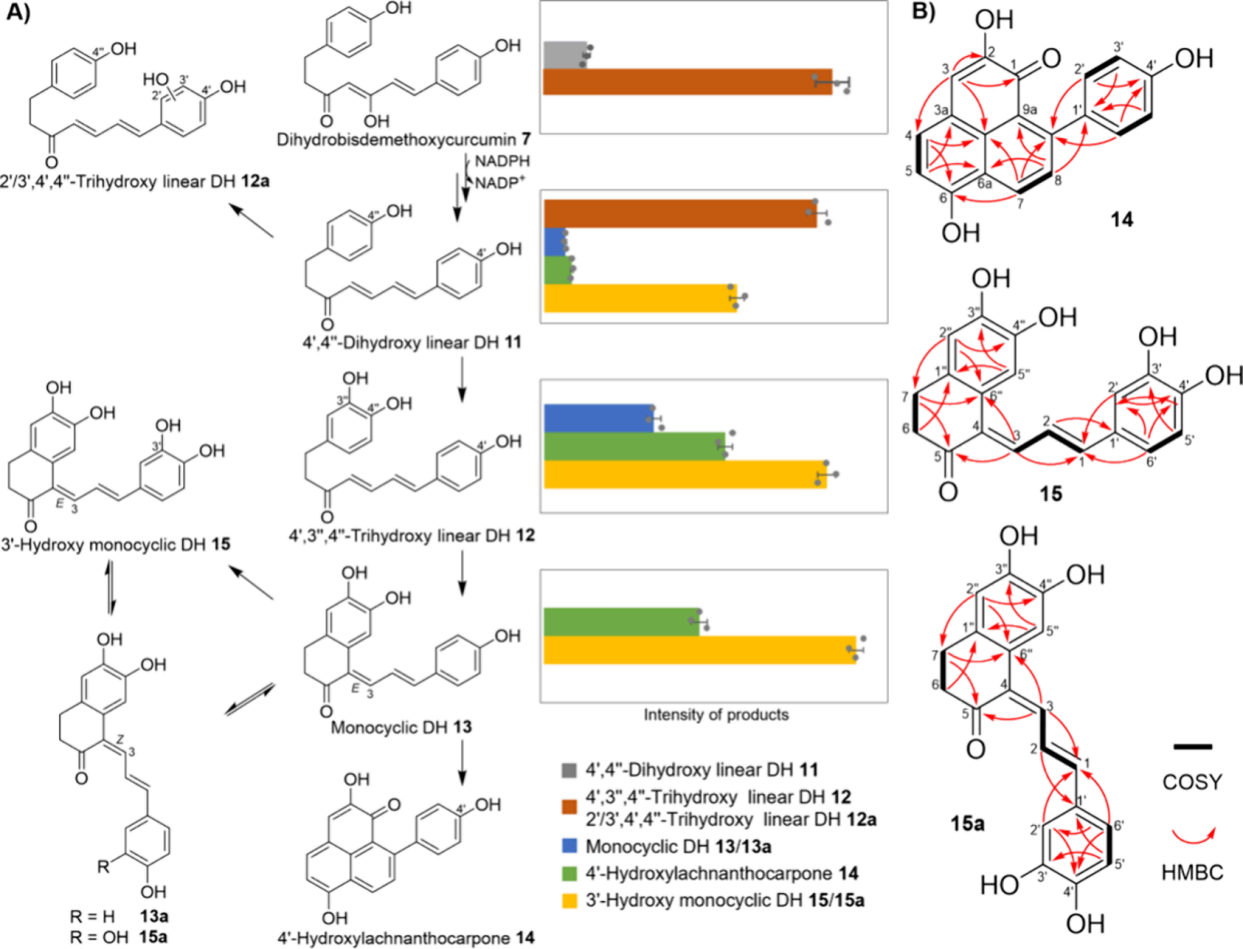
Metabolic conversions catalyzed by protein extracts from *M. lasiocarpa* roots. (A) The substrates **7**, **11**, **12**, and **13** were individually incubated with the crude protein extract. Depicted are the HRESIMS peak areas of products detected in incubations containing the compound immediately to the left. Data are means ± s.e.m. (*n* = 3). All products formed are presented in a unified biosynthetic scheme on the left. Detailed EIC and MS/MS data are shown in Figures S11, S13, S15, and S18. (B) Key COSY and HMBC correlations for **14** and **15**/**15a**.

To detect further downstream conversion steps, isolated **12** was incubated with the root extract giving rise to three unknown [M + H]^+^ ions at *m*/*z* 309.1117, 325.1075, and 305.0806 (calcd for C_19_H_17_O_4_, 309.1121; C_19_H_17_O_5_, 325.1071; C_19_H_13_O_4_, 305.0808) (peaks a–c; Figure S13). NMR analysis revealed two monocyclic diarylheptanoid derivatives **13** and the 3′-hydroxy **15** (Figures S58–S79 and Table S3) and the PP structure 4′-hydroxylachnanthocarpone **14** (Figures S54–S57 and Table S2). Neither **14** nor **15** has been previously characterized.

The idea that monocyclic DHs may be biosynthetic intermediates between linear DHs and PPs was previously raised after a compound related to **13** was isolated from *Fusarium oxysporum*-infected *Musa acuminata*.^7^ Although no biochemical evidence was provided at the time, the authors were able to chemically transform the compound into a PP structure (Figure S14).

To confirm the final cyclization step in PP biosynthesis, isolated **13** and **15** were incubated separately with the crude root protein extract. HRESIMS analysis of assays containing **13** revealed an increase in two peaks with MS/MS signatures corresponding to those of 4′-hydroxylachnanthocarpone **14** and 3′-hydroxy monocyclic DH **15** (peaks a and b; Figure S15). In contrast, no increase of the PP product peaks was detected in incubations with **15**. A possible explanation could be the altered reactivity of **15** caused by the additional hydroxy group at the D-ring.

The identified two-step cyclization of **12** likely proceeds via a sequential 1,4-/1,6-intramolecular Michael addition, initiated by the oxidation of the linear DH substrate to an activated *ortho*-quinone (Figure S16). Given the importance of the keto–enol tautomerized position 6 in the proposed reaction mechanism, it was expected that PP structures without a 6-OH group, which are commonly found in the root extract (Figure S10), are formed in the final step of the pathway. Interestingly, neither the incubation of **14** nor that of any of the upstream intermediates with the crude enzyme extract yielded the expected product, 4′-hydroxyanigorufone **R-1** (Figure S10). This finding may be due to either the limitations of the crude protein extract or the existence of an alternate biosynthetic route to anigorufone-type PPs. The NMR data of **13** and **15** showed that they exist as equilibrium isomeric mixtures, with the Δ^3^ double bond in either *E* (**13** and **15**) or *Z* configuration (**13a** and **15a**, Table S3). The *Z* isomers **13a** and **15a** are likely intermediates in the formation of a group of diarylheptanoids with a rare bicyclic tetrahydropyran motif. Such diarylheptanoids are known constituents of *M. lasiocarpa* and *Musa* × *paradisiaca* (musellarins A–E; Figure S17).^28,29^

The present study provides enzymatic and biochemical insights into the biosynthesis of PPs and linear DH derivatives in *M. lasiocarpa*. Based on the divergent substrate specificities of four type III polyketide synthases and a novel diketide-CoA-accepting medium-chain dehydrogenase/reductase, two distinct assembly lines are responsible for the production of **7** and **8**, the dihydrocurcumin-type precursors of PPs and linear DH derivatives, respectively (Scheme 2A). The stepwise transformation of **7** to 4′-hydroxylachnanthocarpone **14** appears to proceed via two sequential intramolecular Michael additions and constitutes the first complete pathway to a PP scaffold (Scheme 2B).

**Scheme 2:**
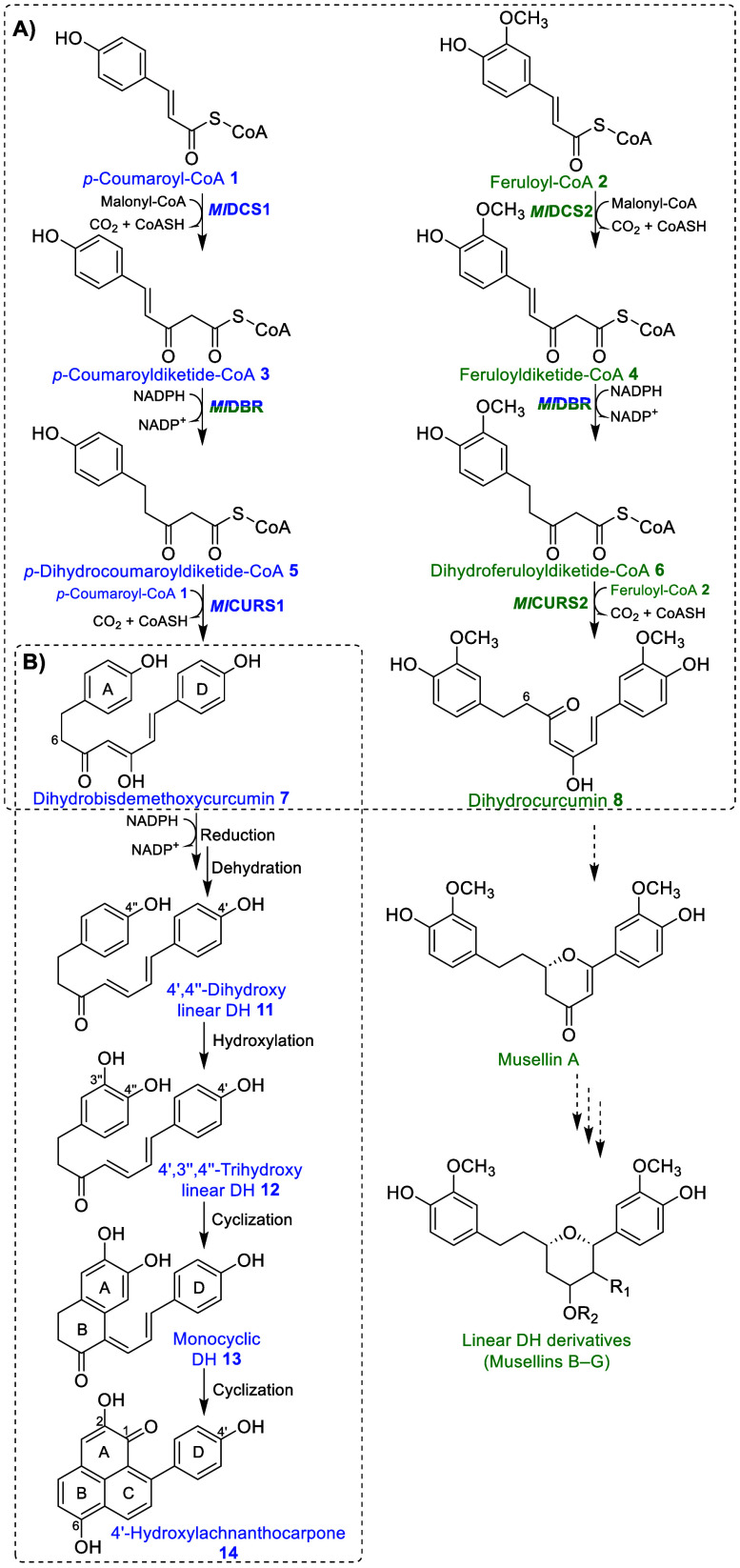
Characterized Conversions in *M. lasiocarpa*: (A) Identified Enzymes Responsible for the Parallel Biosynthesis of **7** and **8**; (B) Downstream Biosynthetic Transformations in Root Protein Extracts

## Data Availability

The data underlying this study are available in the published article and its Supporting Information. Additionally, NMR data of the compounds mentioned in this article can be accessed via https://doi.org/10.17617/3.AKO5SF.

## Supporting Information Available

Materials, experimental details, synthetic procedures, NMR spectra for all isolated and synthetic compounds, additional figures and data including HRESIMS results, extracted ion chromatograms, MS/MS data, and sequence alignments (PDF)
